# Exploring emergent properties in cellular homeostasis using OnGuard to model K^+^ and other ion transport in guard cells^☆^^☆☆^

**DOI:** 10.1016/j.jplph.2013.09.014

**Published:** 2014-05-15

**Authors:** Michael R. Blatt, Yizhou Wang, Nathalie Leonhardt, Adrian Hills

**Affiliations:** a Laboratory of Plant Physiology and Biophysics, University of Glasgow, Bower Building, Glasgow G12 8QQ, UK; b Laboratoire de Biologie du Développement des Plantes, UMR 7265, CNRS/CEA/Aix-Marseille Université, Saint-Paul-lez-Durance, France

**Keywords:** AHA1, plasma membrane proton pump *Arabidopsis* H^+^-ATPase 1, ALMT, aluminum-sensitive malate transporter (gene family), CLC, chloride channel (gene family), [Ca^2+^]i, cytosolic-free calcium concentration, H^+^-ATPase, plasma membrane H^+^ pump, ATP-dependent, H^+^-PPase, vacuolar H^+^ pyrophosphatase pump, H^+^-VATPase, vacuolar H^+^ pump, ATP-dependent, Mal, malate anion, ost2, open stomata 2 protein, identical with AHA1, R-type anion channel, rapid-gating plasma membrane anion channel, ROS, reactive oxygen species, SLAC1, slow anion channel 1 protein (localizes to the plasma membrane), Systems biology, *Arabidopsis*, Guard cell homeostasis, Cytosolic-free [Ca^2+^] and pH, Cytosolic and vacuolar

## Abstract

It is widely recognized that the nature and characteristics of transport across eukaryotic membranes are so complex as to defy intuitive understanding. In these circumstances, quantitative mathematical modeling is an essential tool, both to integrate detailed knowledge of individual transporters and to extract the properties emergent from their interactions. As the first, fully integrated and quantitative modeling environment for the study of ion transport dynamics in a plant cell, OnGuard offers a unique tool for exploring homeostatic properties emerging from the interactions of ion transport, both at the plasma membrane and tonoplast in the guard cell. OnGuard has already yielded detail sufficient to guide phenotypic and mutational studies, and it represents a key step toward ‘reverse engineering’ of stomatal guard cell physiology, based on rational design and testing in simulation, to improve water use efficiency and carbon assimilation. Its construction from the HoTSig libraries enables translation of the software to other cell types, including growing root hairs and pollen. The problems inherent to transport are nonetheless challenging, and are compounded for those unfamiliar with conceptual ‘mindset’ of the modeler. Here we set out guidelines for the use of OnGuard and outline a standardized approach that will enable users to advance quickly to its application both in the classroom and laboratory. We also highlight the uncanny and emergent property of OnGuard models to reproduce the ‘communication’ evident between the plasma membrane and tonoplast of the guard cell.

## Introduction

A major barrier to understanding cellular physiology arises from the complexity of interactions between the transport, metabolic and buffering activities of the different cellular compartments and, at any one membrane, between the assembly of individual transporters that contribute to charge and solute movements across that membrane. Often, the most fundamental question of homeostasis can prove difficult to answer, namely which characteristics arise from these interactions, intrinsic to the assemblies, and which require independent regulatory inputs that engage transcriptional, translational or post-translational modifications. Addressing these and other questions demands a full and quantitative accounting for each contributing transporter, metabolic and buffering reaction, and generally is possible only through integrative modeling to explore the dynamics of these processes within a single ensemble that represents the cell.

In principle, constructing cellular models is not difficult. For many eukaryotic cells, there now exists a substantial body of data that encompasses most, if not all, of the essential parameter sets required for the purpose. However, integrating this information within a systematic mathematical representation can be daunting. Although the essential physicochemical relationships are simple, quantitative functions easily incorporated in a description of the cell, the recursive nature of transport across a common membrane generally defies analytical solution. For each transport process, there exists a unique set of kinetic and regulatory descriptors. However, for a majority of transporters, the process of transport itself acts on one or more of these descriptors. For example, consider the outward-rectifying K^+^ channel of the guard cell. Gating of these channels is sensitive to membrane voltage as well as to K^+^ concentration (Blatt, 1988, 2000b; Blatt and Gradmann, 1997; see also Benito et al., 2014; Demidchik, 2014; Véry et al., 2014). Depolarizing the membrane promotes the current, but the current carried by the channels draws K^+^ out of the cytosol. As a consequence, when otherwise unchecked, the current drives the membrane voltage negative, countering its depolarization and thereby suppressing channel activity. The case of the K^+^ channel is not unique. Every transport process that carries charge across a membrane will affect – and will be affected by – the voltage across that membrane, if only as a consequence of mass action and the movement of the charged ions it carries. In effect, voltage is both substrate and product of charge transport, and is shared between all of the transporters at the membrane. The problem is compounded further because, for charge-carrying transporters operating across a common membrane, these voltage dependencies are frequently non-linear in their characteristics (Blatt and Slayman, 1987; Blatt et al., 1987; Sanders, 1990; Weiss, 1996; Blatt, 2004a). Thus, the challenge becomes one of integrating each and every one of the predominant transporters in a manner that accommodates these recursive and kinetically distinct properties, and of doing so within a system that is sufficiently flexible to allow parameter modifications and substitutions for the equations representing each process between (and even during) modeling sessions.

There are many instances in which modeling has been applied to cellular homeostasis in order to explore potential functions. In plants, for example, this approach has been used effectively to test the feasibility for K^+^ transport to serve as an ‘energy reserve’ for phloem loading of sucrose (Gajdanowicz et al., 2011). However, there are very few instances in which these methods have been applied with sufficient mathematical rigor to yield predictions of unexpected behaviors that have subsequently proven experimentally. Of the latter, dynamic models of mammalian epithelia (Lew et al., 1979) correctly predicted a transient cell shrinkage and protracted fall in tissue short-circuit current following inhibition of the Na^+^/K^+^-ATPase by ouabain. At the time, these experimental findings appeared to contradict the general validity of Ussing's now widely accepted, two-barrier description of mammalian epithelia (Ussing, 1982). The outputs derived from the modeling were counterintuitive, but offered substantive predictions that were confirmed experimentally (MacKnight et al., 1975a,b). More recently, similar models of the erythrocyte demonstrated unexpected connections between hemoglobin metabolism, transport and osmotic balance, both during malarial infection and in sickle-cell anemia, each of which was subsequently verified experimentally (Lew et al., 1991, 2003; Lew and Bookchin, 2005; Mauritz et al., 2009).

Most modeling efforts have been implemented on a case-by-case basis, without a standardized format, and frequently without incorporating the essential transport circuits needed for overall balance of charge, solute and water fluxes (Gradmann et al., 1993; Grabe and Oster, 2001; Shabala et al., 2006; Cui et al., 2009). Utilities such as the Virtual Cell (Loew and Schaff, 2001), E-Cell (Tomita et al., 1999), Cellerator (Shapiro et al., 2003), as well as the Berkeley Madonna Environment (BME) and similar commercial software (Abu-Taieh and El Shiekh, 2007), provide for modeling intra- and intercellular events that encompass reaction-diffusion processes in arbitrary geometries. These utilities offer standardized platforms for modeling, but many are designed for ‘flow-through’ (serial) networks, ill-suited to the recursive nature of membrane transport, and few have the scope to define underlying behaviors, for example as dictated by specific transport equations. Significantly, none of these utilities are flexible in their connections to physiological outputs, most important for plant cells including those of solute content, cell volume, turgor, and non-linear or anisotropic cell expansion. Some of these difficulties are illustrated by the work of Grabe and Oster (2001), who utilized BME software to explore the question of vesicular acidification during endocytosis. Their study was able to recapitulate vesicular pH driven by the V-type H^+^-ATPase, but its prediction of a requirement for a voltage-gated Cl^−^ channel and cation pre-loading was misdirected. Indeed, more recent work has indicated that the Cl^−^ flux essential for vesicular acidification is mediated by CLC-type H^+^–Cl^−^ antiporters (Smith and Lippiat, 2010; Novarino et al., 2010; Weinert et al., 2010).

These limitations are addressed in the Homeostasis, Transport and Signaling (HoTSig) libraries, an approach developed in this laboratory (Hills et al., 2012). HoTSig incorporates an open structure of expandable libraries for transporter kinetics, chemical buffering, macromolecular binding and metabolic reactions, as well as for macroscopic coupling equations such as those relating solute content, cell volume and turgor, all accessible to input and modification by the user. This open structure makes HoTSig adaptable to wide variety of single-cell systems with the potential for its expansion to multicellular situations and problems that must be addressed across scales from the cellular to whole-tissue and organ structures. The first implementation of the HoTSig libraries, in the OnGuard software (Hills et al., 2012; Chen et al., 2012), focused on guard cell mechanics and their control of stomatal aperture. OnGuard (available at www.prsg.org.uk) includes a graphical user interface for real-time monitoring of the individual transport and homeostatic processes under simulation. It incorporates a set of empirically defined equations to relate the output of solute content to cell volume, turgor and stomatal aperture. Finally, it includes a Reference State Wizard as an aid to defining a starting point for experimental simulations with sensible outputs for all known variables. This initial implementation demonstrated that an OnGuard model of the Vicia guard cell recapitulates all of the known characteristics of guard cell transport, solute content and stomatal aperture in the face of well-defined experimental manipulations; it yielded a number of unexpected and emergent outputs, among these a clear demonstration of homeostatic ‘communication’ between the plasma membrane and tonoplast independent of an overlay of control via signal transduction networks; and it demonstrated counterintuitive changes in cytosolic-free calcium concentration ([Ca^2+^]_i_) and pH over the diurnal cycle, all of which find direct support in independent experimental data (MacRobbie, 1991, 1995a, 2000, 2006; Thiel et al., 1992; Blatt and Armstrong, 1993; Willmer and Fricker, 1996; Frohnmeyer et al., 1998; Dodd et al., 2007).

OnGuard models have the power to predict physiology. This capacity is amply demonstrated by the recent study of Wang et al. (2012), who addressed paradoxical observations associated with the *Arabidopsis slac1* mutation. The *slac1* mutant lacks the plasma membrane channel responsible for Cl^−^ loss during stomatal closure (Vahisalu et al., 2008; Negi et al., 2008), but its absence profoundly affects both inward- and outward-rectifying K^+^ channel activities and slows stomatal opening. Analysis of the *Arabidopsis* guard cell using OnGuard predicted the effect to arise from anion accumulation in the mutant, which affects the H^+^ and Ca^2+^ loads on the cytosol, in turn elevating cytosolic pH and [Ca^2+^]_i_, both factors that regulate the K^+^ channels (Wang et al., 2012). These predictions were confirmed experimentally, with the study demonstrating that experimentally ‘clamping’ cytosolic pH and [Ca^2+^]_i_ was sufficient to recover both the K^+^ currents and stomatal opening kinetics. Thus, modeling with the OnGuard software uncovered an entirely unexpected homeostatic network connecting two unrelated ion channels in the guard cell. This study represents a crucial step toward using OnGuard-style modeling to guide the ‘flip side’ problem of reverse-engineering stomatal function; it is a very short step from OnGuard to future implementations that will enable rapid, in silico design as a guide to altering guard cell physiology, for example in improving water use efficiency during photosynthesis (Lawson et al., 2011, 2012; Blatt et al., 2013). Right now, OnGuard can be used to address a wide range of questions in guard cell biology, and further implementations of the HoTSig libraries promise to be equally powerful in enabling research scientists and students to explore similar problems in other plant cells. Here we summarize the elements of an OnGuard model as a guide to users. We provide a didactic review of the *slac1* modeling exercise, and finally, we use OnGuard to explore the *ost2* mutant and address the question of whether suppressing H^+^-ATPase activity at the plasma membrane is essential for stomatal closure and how it affects K^+^ transport, and more generally osmotic solute transport, at both the plasma membrane and tonoplast.

## The elements of an OnGuard model

Stomatal movements arise from the transport, accumulation and release of osmotically active solutes – primarily of K^+^ and Cl^−^, the organic anion malate (Mal) and sucrose – which drive water flux and guard cell turgor (Blatt, 2000b; Schroeder et al., 2001; McAinsh and Pittman, 2009; Hills et al., 2012; Willmer and Fricker, 1996). Predicting stomatal behavior from this wealth of knowledge is generally beyond intuitive grasp, however, and requires a mathematical framework that integrates the transport and metabolism of guard cells to understand stomatal dynamics. In fact, problems of cellular homeostasis of the kind typified by the guard cell are ideally suited to mathematical modeling that integrates the full complement of transport at the major cellular membranes from the ‘bottom and on up.’ Simple, quantitative relations describe the conservation of mass and charge, ion transport and the associated osmotic flux of water, and the membrane voltages that are linked to all ion gradients and permeabilities. These physico-chemical relations constrain all homeostatic models and each is easy to incorporate mathematically. For all plant cells, and especially for the guard cell, the essential homeostatic variables comprise cell volume, cell osmolality, water potential and turgor, membrane potential, the predominant ion concentrations – notably K^+^, Cl^−^ and, to a lesser extent, total and free Ca^2+^ and H^+^ concentrations – as well as the fluxes of these ions and other solutes through each transporter. In turn, these variables are affected by intracellular H^+^ and Ca^2+^ buffering systems, as well as the cell content of impermeant solutes, mostly of protein, their osmotic coefficients, charge and dependence on pH. Quantitative data, including buffering constants, are available for each of the major solutes or can be estimated for endogenous buffer systems (Tsien and Tsien, 1990; Grabov and Blatt, 1997; Tiffert and Lew, 1997; Wang et al., 2012).

Similarly, the biophysical relations of membrane transport are all well defined and, for several plant cell types, including the guard cell, have been studied in sufficient depth for accurate mathematical descriptions. For example, ATP-driven H^+^ pumps, H^+^-coupled transporters and passive ion channels have been characterized, each with regard to stoichiometry and mechanism (Blatt, 1987, 2004b; Sanders, 1990; Blatt et al., 1990a; Willmer and Fricker, 1996), their operation described quantitatively by sets of kinetic equations fully constrained by experimental results. Where knowledge of individual transporters is less well developed, notably at the tonoplast (some insights into vacuolar K^+^ channels are provided in this issue by Ahmad and Maathuis, 2014; Anschütz et al., 2014; Demidchik, 2014; Hamamoto and Uozumi, 2014; Pottosin and Dobrovinskaya, 2014), substantial experimental data, including data on vacuolar ion contents and fluxes, delimit any modeling effort (MacRobbie, 1995b, 2000, 2002; Willmer and Fricker, 1996; Gobert et al., 2007), thereby minimizing the range of parameters that comply with experimental results available for the cell.

Key information is also available for the regulation of transport, including its modulation by [Ca^2+^]_i_ and pH, by reactive oxygen species (ROS; for further aspects on ROS in cellular K^+^ homeostasis see in this issue Anschütz et al., 2014; Demidchik, 2014; Nieves-Cordones et al., 2014; Pottosin and Dobrovinskaya, 2014), and by protein (de-)phosphorylation (Blatt, 2000b; Blatt et al., 2007; Wang and Song, 2008; Kim et al., 2010). Significant mechanistic gaps exist in our understanding of these controls, but are often non-essential: to understand how the system responds to perturbation, the only relevant biology is encapsulated by how one model variable connects to another. This phenomenology is commonly accessible to quantitative experimental analysis, even when the underlying mechanistic details are not. For example, it is well-known that elevated [Ca^2+^]_i_ and protein phosphorylation activate the *slac1* Cl^−^ channel and its homologues in guard cells (Chen et al., 2010), plausibly reflecting the regulation by PYR/PYL/RCAR receptor binding with 2C type protein phosphatases (Geiger et al., 2010, 2011). We do not have sufficient quantitative information to model phosphatase activity, nor its mechanistic connection to [Ca^2+^]_i_ or the Cl^−^ channel. However, the relationship between abscisic acid (ABA), [Ca^2+^]_i_, protein (de-)phosphorylation and channel activation are well established with kinetic detail (Chen et al., 2010). By applying a mathematical description of this relation, we can place the mechanism within a “black box” that subsumes the intermediate kinetics, thus enabling its incorporation within a quantitative model. In general, such ‘black boxes’ effectively parameterize phenomenological modules that greatly reduce complexity and computational burden (Endy and Brent, 2001). A module may be opened when elements subsumed within a module become a target of the modeling and the underlying mechanism(s) can be described in quantitative kinetic detail.

## Defining OnGuard models

Formulating a dynamic model requires an initial or reference condition that represents a physiological baseline from which simulations are then begun. A good place to start is the cell at rest – the guard cell of the closed stoma in the dark, for example – a state in which no net change in solute flux or content occurs. At present, resolving such a reference point, what we referred to as the Reference State and expanded to the Reference Cycle (Hills et al., 2012; Chen et al., 2012), is a laborious process that demands repeated adjustment of model parameters and testing of the model, followed by systematic analysis of model outputs over a range of conditions and their comparison with known experimental data. OnGuard comes with a Reference State Wizard that allows the user to specify the underlying biophysical status of the system and then query the model for the component and net fluxes of each ionic and solute species, as well as parameters for metabolic equilibrium in sucrose and Mal synthesis and catabolism. With the Wizard it is possible to balance each of the components – for example, the fluxes of K^+^ across the tonoplast and plasma membrane – by adjusting the populations of transporters and, if necessary, their underlying kinetic descriptors in order to satisfy the requirements of a Reference State.

Obviously, defining the Reference State implies knowledge of the probable unit densities and/or limiting current amplitudes, as well as the kinetic parameters for each transporter. It is against this knowledge that the biological validity of a model must first be judged to minimize indetermination and ensure its predictive power. There is no hard-and-fast rule as to what is sufficient to ensure predictive power in a model, but there are a few essential guidelines that apply. Most importantly, the pathways for flux of each species must be balanced in both directions across a membrane. For example, a model that incorporates a pathway for the influx of anion X^−^ across the plasma membrane must also include at least one other pathway for its efflux, otherwise flux balance is not possible. For reasons of charge balance, similarly, complementary pathways for oppositely charged species must exist across each membrane. Provided that these conditions are met, and quantitative kinetic detail is available for at least 80–85% of the total flux of all species in both directions and between all compartments, then parameters for the remaining fluxes are usually constrained to a sufficiently narrow range of values relative to one another to render a model with true predictive power. Models based on Reference States and diurnal Reference Cycles for guard cells both of *Vicia* (Chen et al., 2012) and of *Arabidopsis* (Wang et al., 2012) are available for download with the OnGuard software (www.psrg.org.uk) and the full complement of transporters and their parameter sets will be found in the corresponding publications. We recommend starting with these models to circumvent the considerable task of their initial definition. Of course, these ‘pre-packaged’ models come with the standard proviso of all working systems: while the *Vicia* and *Arabidopsis* models we resolved offer good approximations of experimental data, they do so within the bounds of the conditions and data used for validation. No doubt new experimental data will require further refinements to the models and we hope that users will communicate with us for this purpose.

## Simulating the *slac1* mutation of *Arabidopsis*

**Fig. 1:**
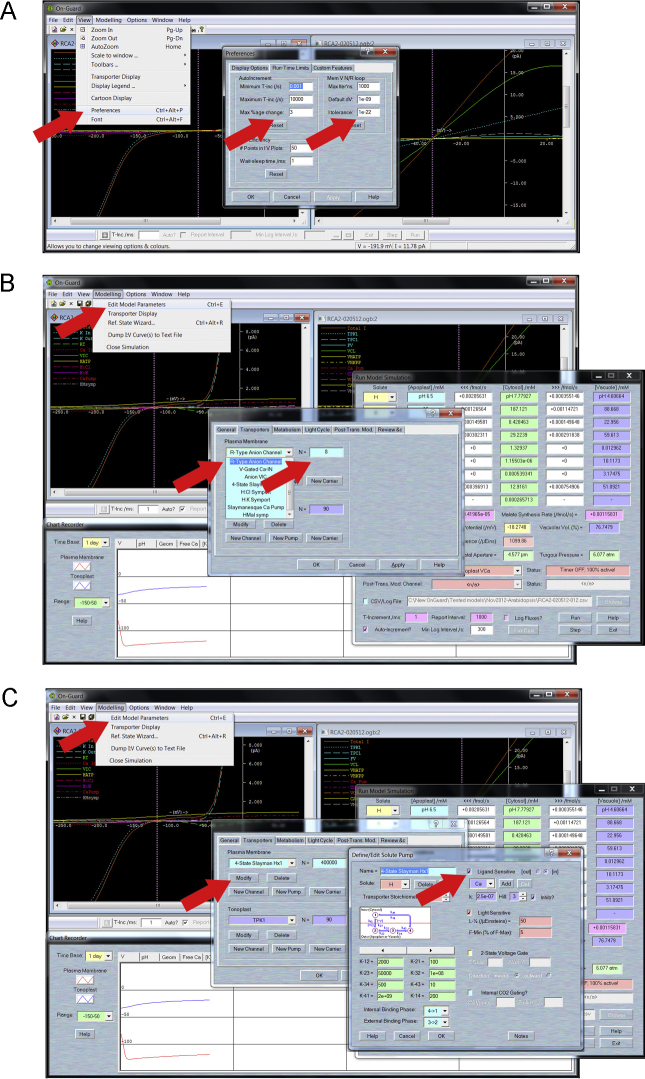
OnGuard screenshots with overlaid dropdown menus, dialog boxes and selections for setting up the runtime limits for calculations (A), for selecting (B) and modifying (C) individual transporters. The selection in (B) is for the R-type anion channel component relevant to the *slac1* simulation, and the dialog boxes in (C) are for the plasma membrane H^+^-ATPase relevant to simulating the *ost2* mutations. Arrows highlight specific selections and actions (see text).

In practice, simulating perturbations that represent new physiological, pathological or experimental conditions is straightforward in OnGuard. To get started, we encourage first-time users to review the 20-min video supplied with the software. This video introduces the basic operation of OnGuard, including, how to access and adjust modeling functions such as temporal minima, maxima and sampling frequencies, how to modify individual transporters, sucrose and malate metabolism, how to change ion and solute concentrations, and how to monitor, interrogate and log OnGuard outputs. Before starting OnGuard for the first time, select ‘View’ in the main window and choose ‘Preferences’ from the drop-down list. In the ‘Run-Time Limits’ tab ensure the ‘Minimum T-inc’ and ‘Maximum T-inc’ values are set to 0.001 and 10,000 s, respectively, the ‘Max %age change’ is set to 2 or 3%, the ‘Max Iterns’ is set to 1000, the ‘Default dV’ is 1 × 10^−9^ and the ‘I tolerance’ is 1 × 10^−22^ (Fig. 1A, arrows). You can adjust the ‘O/P Efficiency’ settings for the best compromise between speed and quality in graphical display. Increasing the points plotted per curve increases the display burden on the processor, while increasing the ‘Wait-sleep time’ slows the computational cycle to allow time for display functions.

We recommend setting the software to ‘Auto-increment’ time (tickbox at the lower left of the flux window) and the adjacent ‘Min Log Interval’ to 20 s for better temporal resolution. For comparative purposes, run a model, in this case the *Arabidopsis* guard cell model supplied with the software, through the equivalent of three diurnal cycles (72 h) as a control. Shortly before the end of 72 h, pause the simulation (click the ‘Stop’ button on the control panel or at the bottom right of the flux window). Do not close any windows, as this will terminate the simulation and close the data log. Use the ‘Modelling’ drop-down menu at the top of the main window, select ‘Edit Model Parameters’ and then the ‘Transporters’ tab. Choose the ‘Anion VIC’ in the Plasma Membrane drop-down list and enter 0 (zero) for the value ‘*N*’ (the scaled population size of this transporter/characteristic) to the right of the list (Fig. 1B, arrows); then select R-type anion channel from the drop-down list and enter an ‘*N*’ value of 2. [For computational simplicity, we incorporated the *slac1* characteristics as the sum of a voltage-dependent component and a voltage-independent component (Wang et al., 2012; Hills et al., 2012). These new settings are equivalent to eliminating *slac1* from the model. The remaining ‘R-Type Anion Channel’ population represents the ALMT component current.] Resume the simulation by clicking the ‘Run’ button on the control panel or at the bottom right of the flux window. Again, we recommend that OnGuard run with these new settings for at least three, full diurnal cycles to ensure a new stable cycle is achieved. Finally, at the end of this period the simulation can be paused again, the values of ‘*N*’ for the two *slac1* components reset to their starting values, and the simulation resumed for a further three or four diurnal cycles. Terminate the simulation by clicking the ‘Stop’ button and then close the flux window, which also closes the *.csv file log.

OnGuard normally logs the contents of each compartment (apoplast, cytosol, vacuole) and the net fluxes across each membrane (plasma membrane, tonoplast) for each ionic and solute species, as well as [Ca^2+^]_i_ and the rates of sucrose and malic acid synthesis. Many of these variables are also available during simulations via an on-screen chart recorder. Users can choose to log the fluxes of each ionic species through each of the different transporters individually at the two membranes. All data are logged in *.csv format, which is readable by most spreadsheet programs. For a first review of the OnGuard output, importing and plotting the data using Microsoft Excel is ideal. For more detailed analysis and for generating publication-quality graphs, we use the Systat software package SigmaPlot. Interpreting the outputs then becomes a matter of interrogating the model variables. Just as *in vivo*, changes in each of these variables – including the various solute concentrations, membrane voltages, cytosolic-free [Ca^2+^] and pH, as well as the rates of ion and solute flux through each of the transporters – arise through interactions between the transporters, metabolism and associated buffering characteristics. These variables are commonly the most helpful to identifying the emergent behaviors of the system as a whole and interpreting their origins. Even a brief comparison of the output with the control (wild-type) and *slac1* parameters shows substantial effects on stomatal aperture and its dynamic range over the diurnal cycle, as well as substantial increases in the K^+^, Cl^−^ and malate contents of the cytosol and vacuole. These outputs can be found in Fig. 4 and the Supplemental figures of Wang et al. (2012). A detailed explanation for the various effects of the mutant can be found in the legends to these Supplemental figures, so a few brief observations will suffice here. Most important, the effect of *slac1* in retaining K^+^ can be ascribed principally to a negative shift in the free-running voltage at the plasma membrane (and *vice versa* at the tonoplast) such that the time-averaged driving force for K^+^ flux favors its retention [see Supplemental Fig. S1 of Wang et al. (2012)].

OnGuard predicts a substantial inactivation of the inward-rectifying K^+^ current and activation of the outward-rectifying K^+^ current in *slac1*. The current–voltage curves for each of these currents [see Fig. 4C and D of Wang et al. (2012)] are accessible for export from OnGuard. Simply pause the simulation at the same relative time of day when running with the control, and then with the *slac1* parameters, use the ‘Modelling’ drop-down menu and select ‘Dump I:V Curve(s) to Text File’ to export these data before importing and plotting either using Excel or another graphical software package. The reduced current at the free-running plasma membrane voltage accounts for the slower K^+^ uptake, despite the effect in hyperpolarizing the membrane of *slac1* elimination. Similarly, the relative membrane hyperpolarization at the end of the day in *slac1* [see Supplemental Fig. S1 of Wang et al. (2012)] counters the effect of the mutant in increasing the capacity for K^+^ efflux through the outward-rectifying K^+^ channels [Fig. 4C of Wang et al. (2012)]. How does the *slac1* mutant affect these two, wholly unrelated K^+^ channels? The answer lies in the OnGuard prediction of elevated cytosolic pH and [Ca^2+^]_i_ in the mutant [Fig. 4E of Wang et al. (2012)], both of which suppress the inward K^+^ current while the more alkaline pH enhances the outward K^+^ current (Blatt et al., 1990b; Blatt and Armstrong, 1993; Lemtiri-Chlieh and MacRobbie, 1994; Miedema and Assmann, 1996; Grabov and Blatt, 1997, 1999). The rise in [Ca^2+^]_i_ [see Fig. 4E and Supplemental Fig. S6A of Wang et al. (2012)] can be traced directly to plasma membrane hyperpolarization in *slac1* [see Supplemental Fig. S1 of Wang et al. (2012)] and the consequent increase in Ca^2+^ influx across the plasma membrane [see Supplemental Fig. S6D of Wang et al. (2012)]. The rise in cytosolic pH [see Fig. 4E and Supplemental Fig. S4A of Wang et al. (2012)] arises primarily from Cl^−^ and malate accumulation, which act to trans-inhibit H^+^-coupled Cl^−^ transport across the tonoplast and plasma membrane and malic acid synthesis [see Supplemental Figs. S3 and S4 of Wang et al. (2012)]. Finally, it is worth noting that eliminating this one channel at the plasma membrane has profound effects on transport at the tonoplast, not just on Cl^−^ and malate transport, but also on vacuolar Ca^2+^ [see Supplemental Fig. S6 of Wang et al. (2012)] and K^+^ transport [see Supplemental Fig. S7 of Wang et al. (2012)]. While the latter observations of changes in K^+^ and Ca^2+^ flux at the tonoplast remain to be confirmed experimentally, they are implicit in the published characteristics of the *slac1* mutant (Vahisalu et al., 2008; Negi et al., 2008; Wang et al., 2012); most important, they serve to underline our earlier observation of homeostatic communication between the plasma membrane and tonoplast that emerges from the intrinsic properties of the several transporters at the two membranes.

## The *ost2* mutation of *Arabidopsis* and its implications for stomatal movements

Guard cells of *Arabdopsis* express primarily three of the eleven H^+^-ATPase (AHA) genes encoded within the genome (Lopez-Marques et al., 2004). Of these, AHA1 is the most highly expressed; it was previously identified with two dominant (*ost2*) mutations encoding single site residue substitutions that confer enhanced and constitutive activity in H^+^-ATPase activity and insensitivity to the water stress hormone ABA (Merlot et al., 2007). The *ost2* mutants have proven similarly unable to fully close stomata in the presence of bacterial pathogens, which normally gain entry to the leaf through the stomatal pore (Liu et al., 2009). The effect of ABA on net H^+^-ATPase activity is not known, but has been suggested to reduce its total activity in guard cells by some 65% (Roelfsema et al., 1998; Zhang et al., 2004). Nonetheless, whether H^+^-ATPase inhibition is important for stomatal closure remains contentious, and it has been argued forcefully that activating guard cell anion channels is sufficient to drive closure without any change in the H^+^-ATPase (Levchenko et al., 2005; Kim et al., 2010).

Significantly, both *ost2* mutants show supra-optimal stomatal apertures and remain partially responsive to light–dark transitions, but are virtually insensitive to elevated Ca^2+^ outside (Merlot et al., 2007). Raising external Ca^2+^ is known to affect [Ca^2+^]_i_, and the association suggests a loss in Ca^2+^-dependence of the H^+^-ATPase (Kinoshita et al., 1995) may be an important consequence of the mutations. Thus, as a first approximation for the effects of the *ost2* mutation, we can eliminate the H^+^-ATPase sensitivity to [Ca^2+^]_i_. Let us use this assumption as the basis to test the opposing interpretations of the H^+^-ATPase contribution to closing using the OnGuard *Arabidopsis* model. Of course, there are other ways to simulate the effects of *ost2* on H^+^-ATPase activity. For example, it might postulated that the H^+^-ATPase is uncoupled from regulation by light, and hence from ATP turnover (Chen et al., 2012). In either case, the question to be asked is whether inhibition of the pump is essential for stomatal closure. Obviously, modeling the *ost2* mutations through the [Ca^2+^]_i_-sensitivity of the pump explores whether this one change in the H^+^-ATPase properties is sufficient to reproduce the mutant characteristics. Most important, we can investigate the output of the OnGuard *Arabidopsis* model to examine what experimentally testable predictions arise from the simulation.

We have carried out the simulation, uncoupling H^+^-ATPase activity from [Ca^2+^]_i_, and encourage others to do the same. Again, run the model through the equivalent of three diurnal cycles, 72 h of simulation time, as a control. Shortly before the end of the 72 h, pause the simulation (click the ‘Stop’ button on the control panel or at the bottom right of the flux window) without closing any windows. Use the ‘Modelling’ drop-down menu at the top of the main window, select ‘Edit Model Parameters’ and then select the ‘Transporters’ tab. Choose the ‘4-state Slayman Hx1′ in the Plasma Membrane drop-down list, then click ‘Modify’ immediately below the list entry. [This transporter corresponds to the H^+^-ATPase as described by a 4-state carrier cycle (visible in the editing window) with reaction constant values that are drawn from analyses of the *Chara* and *Vicia* guard cell H^+^-ATPases (Blatt, 1987; Blatt et al., 1990a).] Click on the tick box on the upper right to deselect the ‘Ligand-sensitive’ parameters for Ca^2+^ (Fig. 1C, arrows). Finally, click ‘OK’ at the bottom of the window and again at the bottom of the ‘Transporters’ tab window. The H^+^-ATPase is now set to operate independent of any changes in [Ca^2+^]_i_. Resume the simulation by clicking the ‘Run’ button on the control panel or at the bottom right of the flux window. Again, run OnGuard with these new settings for at least three, full diurnal cycles to ensure a new stable cycle is achieved. Finally, at the end of this period the simulation can be paused again, the ‘Ligand-sensitive’ parameters for [Ca^2+^]_i_-sensitivity reinstated by clicking the tick box back on, and the simulation resumed for a further three or four diurnal cycles before terminating the simulation by clicking the ‘Stop’ button and closing the flux window.

**Fig. 2:**
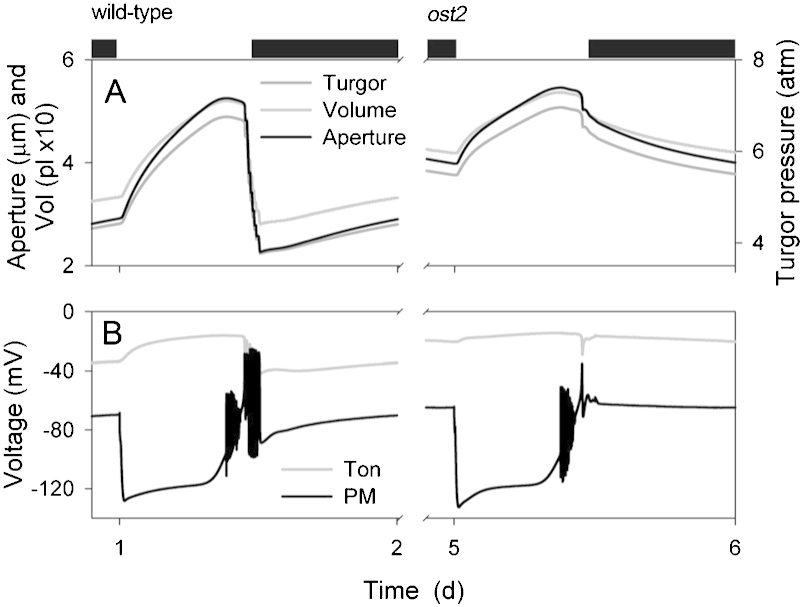
Macroscopic outputs from the OnGuard model. Outputs resolved over a standard diurnal cycle (12 h light:12 h dark, indicated by bars above) with 10 mM KCl, 1 mM CaCl2 and pH 6.5 outside (Chen et al., 2012). Representative diurnal cycles are shown for the wild-type (left) and the *ost2* mutant (right). For the results in this and the subsequent figures, the simulation was carried out as described in the text, first with wild-type parameters. After 72-h simulation time, the *ost2* mutation was introduced by eliminating H^+^-ATPase sensitivity to [Ca^2+^]_i_. A summary analysis is provided with each of the subsequent figures; further details will be found in Chen et al. (2012). The full set of model parameters and initializing variables are listed in Wang et al. (2012) and are available with the OnGuard software at www.psrg.org.uk. Shown are (A) stomatal aperture, turgor pressure and total guard cell volume, and (B) plasma membrane and tonoplast voltage.

**Fig. 3:**
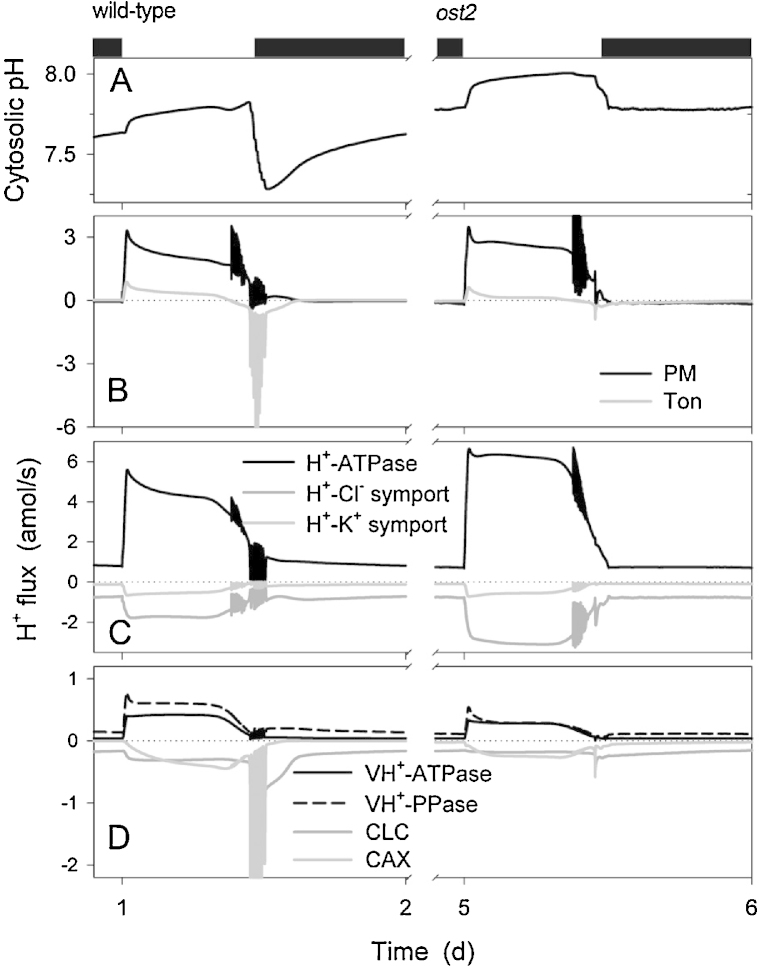
Cytosolic pH and analysis of H^+^ fluxes across the plasma membrane and tonoplast. Outputs resolved over the standard diurnal cycle (12 h light:12 h dark, indicated by bars above) (Chen et al., 2012) for the wild-type (left) and the *ost2* mutant (right). Shown are (A) cytosolic pH, (B) the net H^+^ flux across the plasma membrane and tonoplast, (C) the H^+^ flux through the H^+^-permeable transporters at the plasma membrane, (H^+^-ATPase, and the H^+^–K^+^ and H^+^–Cl^−^ symporters), and (D) the H^+^ flux through the H^+^ permeable transporters at the tonoplast (VH^+^-ATPase, VH^+^-PPase, the CLC H^+^–Cl^−^ antiporter and the CAX H^+^–Ca^2+^ antiporter). Positive flux is defined as movement of the ionic species (not the charge) out of the cytosol, either across the plasma membrane or the tonoplast. The OnGuard model anticipated a substantial time-averaged rise in cytosolic pH in the *ost2* mutant compared with in the wild-type, and an increase in H^+^-ATPase transport which, to an extent was compensated by roughly a 2-fold rise in H^+^ return via H^+^-coupled Cl^−^ transport. Note that the *ost2* mutant also showed a reduction in H^+^ transport to the vacuole via the H^+^-VATPase and the H^+^-PPase as well as H^+^ return, notably via the CAX H^+^–Ca^2+^ antiporter (D).

**Fig. 4:**
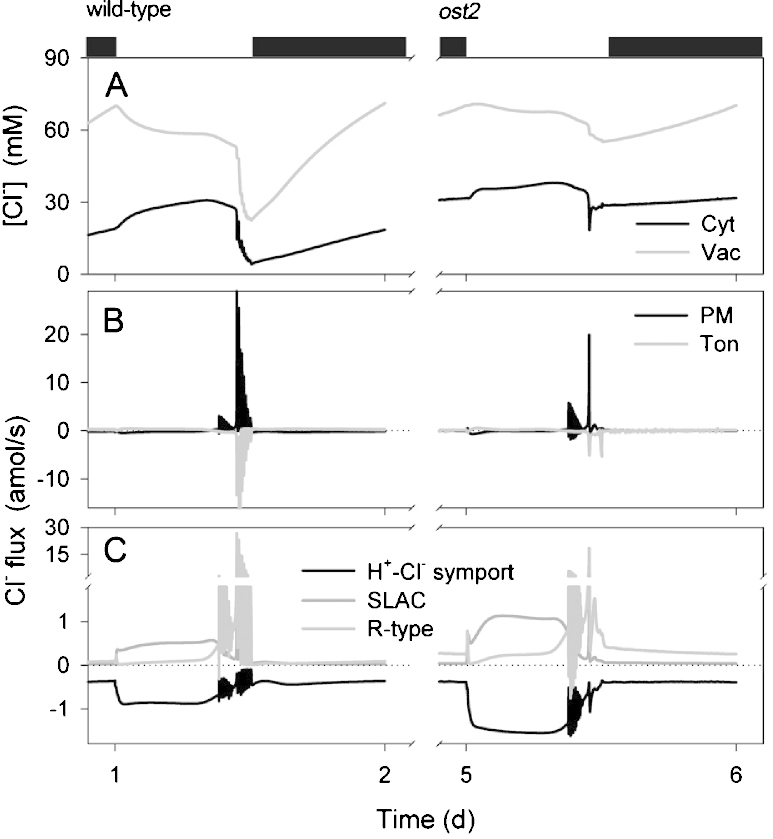
Chloride contents and analysis of Cl^−^ fluxes at the plasma membrane and tonoplast. Outputs resolved over a standard diurnal cycle (12 h light:12 h dark, indicated by bars above) (Chen et al., 2012) for the wild-type (left) and the *ost2* mutant (right). Shown are (A) total cytosolic and vacuolar [Cl^−^], (B) the net flux of Cl^−^ across the plasma membrane and tonoplast, and (C) the flux of Cl^−^ through the Cl^−^-permeable transporters at the plasma membrane (SLAC and R- (ALMT-) type anion channels and H^+^–Cl^−^ symporter). Again, positive flux is defined as movement of the ionic species (not the charge) out of the cytosol, either across the plasma membrane or the tonoplast. Note the elevation in [Cl^−^] in both the cytosol and vacuole, and the loss in their dynamics in the *ost2* mutant. Closure was marked by a large flux of Cl^−^ from the vacuole to the cytosol and export across the plasma membrane, but the pattern was lost in the mutant, reflecting the loss of cyclic changes in voltage and [Ca^2+^]_i_ that drive solute efflux from the vacuole and across the plasma membrane [see Chen et al. (2012)].

**Fig. 5:**
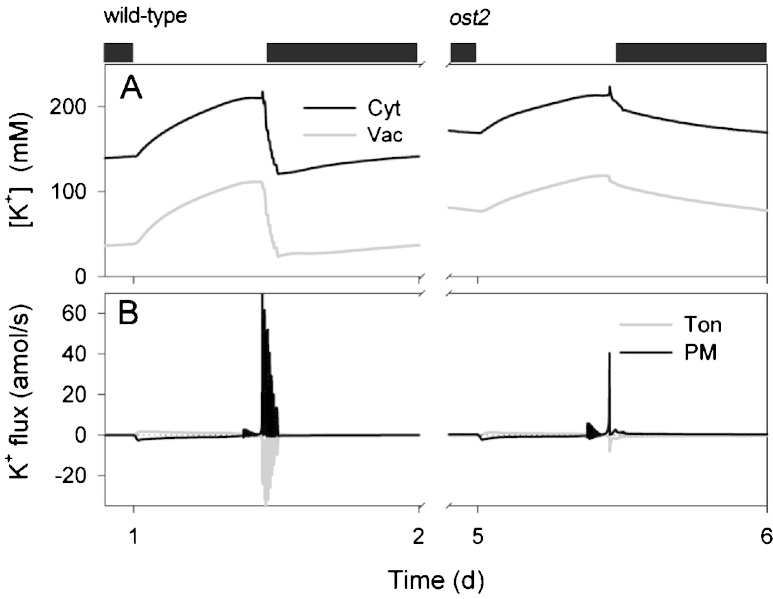
K^+^ contents and analysis of K^+^ fluxes at the plasma membrane and tonoplast. Outputs resolved over a standard diurnal cycle (12 h light:12 h dark, indicated by bars above) (Chen et al., 2012) for the wild-type (left) and the *ost2* mutant (right). Shown are (A) cytosolic and vacuolar [K^+^], and (B) the net K^+^ flux across the plasma membrane and tonoplast. In the wild-type, the cytosolic K^+^ concentration varied between approximately 140 mM and 230 mM; in the vacuole, K^+^ concentrations ranged between approximately 40 and 120 mM (A). The major proportion of K^+^ entering across the plasma membrane was shunted across the tonoplast to the vacuole during the day and this pattern reversed in the first hours of dark, as expected from experimental observation (Hills et al., 2012; Chen et al., 2012). As is the case for Cl^−^, the model predicted the *ost2* mutant to retain substantial levels of K^+^ in both cytosol and vacuole, with diurnal variations evident over a reduced range (A). Flux of K^+^ into the vacuole early in the daylight period was slowed in *ost2* compared to the wild-type and efflux from the vacuole at the end of the day was greatly suppressed (B), reflecting the reduced electrical driving force for K^+^ transport out of the vacuole and the loss of cyclic changes in voltage and [Ca^2+^]_i_ that drive solute efflux from the vacuole and across the plasma membrane [see Chen et al. (2012)].

**Fig. 6:**
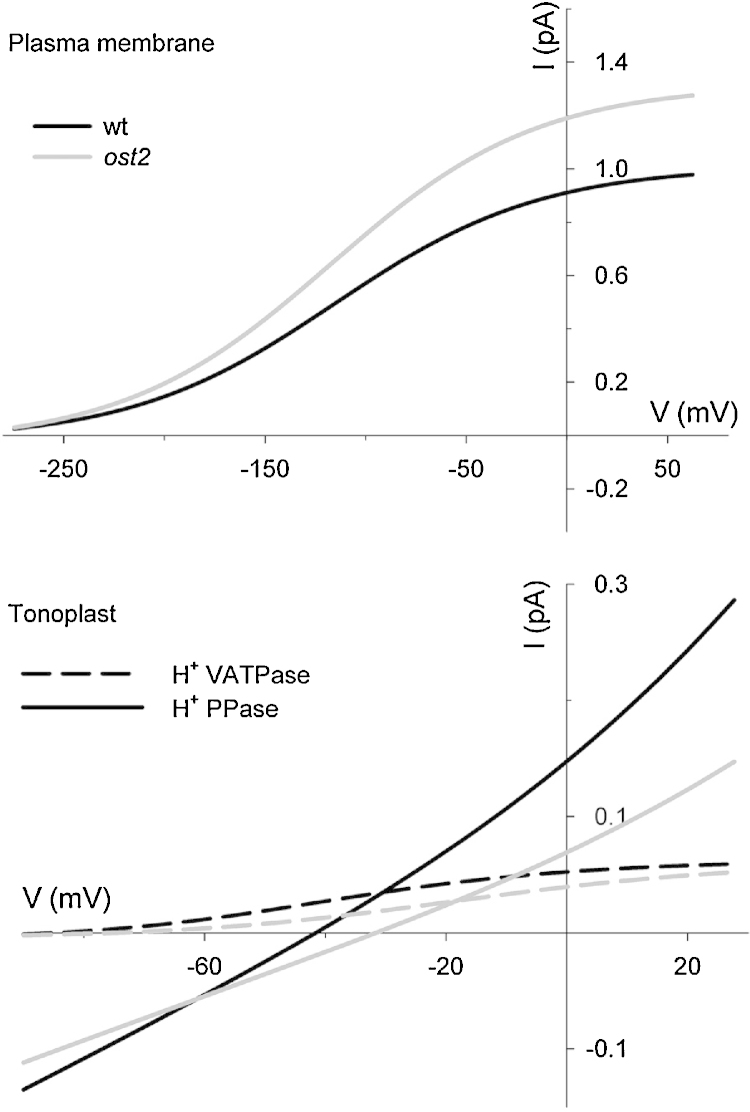
Current–voltage curves for the plasma membrane H^+^-ATPase (above) and the tonoplast H^+^-VATPase and H^+^-PPase (below) taken at 2 h into the light period in each case. Curves for the *ost2* mutant are shown in gray. At the free-running voltage, the model predicts roughly a 20% increase in H^+^-ATPase current at the plasma membrane, despite the reduced substrate (H^+^) concentration. It also predicted roughly a 50% decrease in H^+^ transport into the vacuole at the free-running voltage via the two tonoplast pumps in *ost2*.

A selection of our results is summarized in Figs. 2–5 and shows a diurnal cycle from the wild-type (control) and *ost2* mutant conditions described above and extracted from the logged data once a stable cycle was established. Fig. 6 illustrates the simulated vacuolar H^+^-VATPase and H^+^-PPase current characteristics taken at 2 h into the light period for each of these cycles. The first and most obvious effect of eliminating the [Ca^2+^] is sensitivity of the H^+^-ATPase was that stomatal aperture is elevated, opening and closure are both slowed, the dynamic range of apertures is reduced, and the free-running membrane voltage is hyperpolarized (Fig. 2), much as was reported by Merlot et al. (2007). Simulation with the wild-type parameters yielded rapid, almost step-wise closure that is associated with extended oscillations in [Ca^2+^]_i_, membrane voltage, K^+^ and anion channel activities (Chen et al., 2012), much as has been inferred from past studies with ABA and other closing stimuli (Blatt and Armstrong, 1993; Blatt and Thiel, 1994; McAinsh et al., 1995; Blatt, 2000a; Yang et al., 2006). At the end of the day, closure is initiated by a proportional reduction in all ATPase activities (Chen et al., 2012), and is potentiated by cycles of [Ca^2+^]_i_ elevation, which promote flux through the anion channels and outward-rectifying K^+^ channels while inactivating the inward-rectifying K^+^ channels at the plasma membrane (Blatt, 2000b; Chen et al., 2012). When run with the putative *ost2* mutant parameters; however, these oscillations are suppressed, bar an initial, abortive depolarization, small decrease in aperture (Fig. 2) and the baseline reduction in ATPase activity at the end of the day (Fig. 3). The remaining reduction in aperture is evident as a slow decay that persists throughout the dark period and culminates in a minimum aperture close to the maximum value observed with the wild-type parameters. Clearly, then, a challenge mid-day with ABA or another stimulus that elevates [Ca^2+^]_i_ will have even less effect on stomatal aperture when the H^+^-ATPase is not constrained by the day-end. Additionally, the model showed a substantial increase in H^+^ export across the plasma membrane via the H^+^-ATPase and elevation of cytosolic pH in the *ost2* mutant compared to the wild-type over the entire diurnal cycle (Fig. 3). With the exception of cytosolic pH which has yet to be determined, these characteristics mirror experimental observations (Merlot et al., 2007). They are entirely consistent with the enhanced activity of the H^+^-ATPase when unfettered from the normal restrictions of [Ca^2+^]_i_. In short, by the simple expedient of uncoupling H^+^-ATPase activity from its regulation by [Ca^2+^]_i_, we are able to reproduce a complete set of phenotypic characteristics that closely match those of the *ost2* mutant.

Of course, the utility of any homeostatic model lies not only in its ability to recapitulate physiological behavior but, more important, in its capacity to make experimentally testable predictions. Several can be drawn from these simulations, so suffice it to highlight a few of the most obvious here. Bear in mind that experimentally validating these predictions would support the assumption that the *ost2* mutants affect the [Ca^2+^]_i_ is sensitivity of the pump. First and foremost, modeling the *ost2* mutant predicts a substantial retention of Cl^−^ in the vacuole, and to a lesser extent in the cytosol, throughout the diurnal cycle (Fig. 4). It also predicts the enhanced transport of Cl^−^ across the plasma membrane (Fig. 4) and an overall reduction in H^+^ transport into the vacuole via the H^+^-VATPase and H^+^-PPase activities (Figs. 3 and 6). Transport of Cl^−^ is enhanced across the plasma membrane, despite the increase in Cl^−^ concentrations both in the cytosol and vacuole, and can be attributed to the favorable H^+^ electrochemical gradient across the plasma membrane. The time-averaged increase in K^+^ concentrations mirrors these characteristics (Fig. 5). The retention of these solutes can ultimately be attributed to the loss in cyclic Ca^2+^ influx at the plasma membrane and [Ca^2+^]_i_ elevations during closure, which are essential to facilitate osmotic (K^+^, Cl^−^, malate) flux across the tonoplast and promote CAX-mediated H^+^ influx through Ca^2+^–H^+^ exchange across the tonoplast (Chen et al., 2012). In short, although the primary target of the *ost2* mutation rests in the activity of the H^+^-ATPase at the plasma membrane, these simulations once again underline the homeostatic communication with the vacuole and suggest the most profound effects on transport at the tonoplast.

## Conclusion and outlook

From the simulations illustrated here, two general observations can be drawn. The first – that transporters interact when operating at one membrane – is implicit in the nature of transport, which must occur in parallel across a common membrane, and was highlighted at the start of this article. It arises from the commonality of membrane voltage, and in many cases also the pools of ionic species on either side of the membrane, and it leads to both direct and indirect interactions between otherwise unrelated transporters. The example of *slac1* is a case in point in which eliminating a Cl^−^ channel results in substantial changes in the intrinsic activities of two different K^+^ channels at the same membrane. The reader will discover similar patterns with the *ost2* mutant simulations outlined here, and in many other situations. The second observation similarly arises from the commonality of the pools of ionic species that are transported across the serial barriers of the plasma membrane and tonoplast. In this case, the resulting coordination between fluxes of each ionic species at the two membranes leads to an uncanny sense of ‘communication’ between membranes. Yet this emergent behavior arises simply from the interconnections between [Ca^2+^]_i_ and pH as well as the shared ‘substrates’ and ‘products’ that comprise the various solutes transported across these membranes.

In hindsight, it should be obvious that the consequence of manipulating a single transporter at a membrane is rarely (if ever) restricted to this one process or solely to the distributions of the transported species. The difficulty is in anticipating the consequences of such manipulations, because they are generally beyond intuitive grasp. Clearly, these problems can only be addressed satisfactorily through quantitative mathematical modeling, such as the modeling illustrated here. We anticipate that the HoTSig libraries, on which the OnGuard software is built, will find applications in exploring many other cell systems for which there is kinetic detail of transport sufficient to develop truly predictive models. The modular construction of the HoTSig libraries (Hills et al., 2012) means that solute content, volume and turgor can be ‘bolted on’ to any phenomenological descriptors for virtually any plant cell type.
